# Is No. 12a Lymph Node Dissection Compliance Necessary in Patients Who Undergo D2 Gastrectomy for Gastric Adenocarcinomas? A Population-Based Retrospective Propensity Score Matching Study

**DOI:** 10.3390/cancers15030749

**Published:** 2023-01-25

**Authors:** Yun-Feng Zhu, Kai Liu, Wei-Han Zhang, Xiao-Hai Song, Bo-Qiang Peng, Xu-Liang Liao, Xiao-Long Chen, Lin-Yong Zhao, Kun Yang, Jian-Kun Hu

**Affiliations:** Gastric Cancer Centre, Department of General Surgery, West China Hospital of Sichuan University, Chengdu 610041, China

**Keywords:** gastric cancer, lymph node dissection, D2 gastrectomy

## Abstract

**Simple Summary:**

Since nodal metastasis is the main pattern for gastric cancer (GC) metastasis, lymph node (LN) dissection is essential for accurate staging and improving prognosis. However, debates exist regarding the necessity of No. 12a LN dissection (LND) in D2 gastrectomy. Moreover, the compliance rate for No. 12a LND in practice is low. To explore No. 12a LND noncompliance’s effect on long-term prognosis in GC patients after D2 gastrectomy, we performed a retrospective propensity score matching study with 2788 patients included. The results showed that patients with No. 12a LND had a significantly greater OS than those without it before and after PSM. This study is the first propensity score matching study to demonstrate the prognostic impact of No. 12a LND noncompliance on patients who undergo D2 gastrectomy. This large population-based study may provide guidance on No. 12a LND.

**Abstract:**

LN dissection is essential for accurately staging and improving GC patient prognosis. However, the compliance rate for No. 12a LND in practice is low, and its necessity is controversial. Data from GC patients who underwent total gastrectomy (TG)/distal gastrectomy (DG) plus D2 lymphadenectomy between January 2000 and December 2017 at West China Hospital, Sichuan University were reviewed. No. 12a LND noncompliance’s effect on the long-term prognosis of patients with GC after D2 gastrectomy was explored. Of the 2788 patients included, No. 12a LND noncompliance occurred in 1753 patients (62.9%). Among 1035 patients with assessable LNs from station 12a, 98 (9.5%) had positive LNs detected at station 12a. No. 12a LN metastasis patients (stage IV not included) had significantly better overall survival (OS) than TNM stage IV patients (*p* = 0.006). Patients with No. 12a LND compliance had a significantly higher OS than those without, both before (*p* < 0.001) and after (*p* < 0.001) PSM. Cox multivariate analysis confirmed that No. 12a LND noncompliance was an independent prognostic factor before (HR 1.323, 95% CI 1.171–1.496, *p* < 0.001) and after (HR 1.353, 95% CI 1.173–1.560, *p* < 0.001) PSM. In conclusion, noncompliance with No. 12a LND compromised the long-term survival of patients who underwent D2 gastrectomy for GC.

## 1. Introduction

Gastric cancer (GC) is the fifth most common malignant tumor and the fourth leading cause of cancer-related death worldwide [1]. Surgical resection remains the mainstream treatment for GC, especially for locally advanced GC [2,3]. As nodal metastasis is the main pattern for GC metastasis, lymph node (LN) dissection is essential for accurate staging and improving prognosis. D2 lymphadenectomy, proposed by Japanese scholars, has been the consensus treatment for patients with locally advanced GC [3,4,5].

The hepatoduodenal ligament LNs were subdivided into No. 12a (hepatoduodenal ligament LNs along the proper hepatic artery), No. 12b (hepatoduodenal ligament LNs along the bile duct) and No. 12p (hepatoduodenal ligament LNs along the portal vein) by the Japanese Gastric Cancer Association (JGCA) [6]. Station 12a LNs have been included in D2 lymphadenectomy by the JGCA due to their high therapeutic index [7]. However, the seventh edition of the *Cancer Staging Manual of the American Joint Committee on Cancer (AJCC)* once classified hepatoduodenal LN metastasis as distant metastasis because of its poor prognosis [8].

Debates exist regarding the necessity of No. 12a LN dissection (LND) in D2 gastrectomy. Some studies suggested that stage I-III patients with No. 12a LN metastasis had a better prognosis than stage IV patients [9,10,11]. Thus, hepatoduodenal LNs were reclassified as regional LNs in the eighth edition of the *Cancer Staging Manual of AJCC* [12]. However, some studies have suggested that the incidence of No.12 LN metastasis seems relatively low, and No. 12a LND noncompliance does not compromise long-term survival [13,14]. Currently, the dissection of station 12a LNs has not been emphasized in the National Cancer Comprehensive Network (NCCN) guidelines for gastric cancer (Version 2, 2022) [15].

Another noteworthy fact is the low compliance rate for No. 12a LND in actual practice. A recent Korean real-world study reported an extraordinarily low compliance rate for No. 12a LND (only 29%) [14]. Another study from China reported that noncompliance with No. 12a LND occurred in nearly half of total gastrectomy (TG) patients and more than one-third of the distal gastrectomy (DG) patients [16]. Moreover, European scholars also found that D2 noncompliance mainly involved nodal station 12a in both TG and DG [17].

Therefore, this study aimed to clarify whether noncompliance with No. 12a LND in D2 resection for GC compromises long-term survival and to investigate which patients would benefit from No. 12a LND.

## 2. Materials and Methods

### 2.1. Data Resources and Ethical Standards

Data from this study were collected from the Surgical Gastric Cancer Patient Registry of West China Hospital (WCH-SGCPR). The establishment of WCH-SGCPR was approved by the Research Ethics Committee of West China Hospital, Sichuan University (Register number WCHSGCPR-2018-09). All medical records from WCH-SGCPR were anonymized during the analysis. Informed consent of patients was waived because of the retrospective nature of this study.

### 2.2. Study Population

Data from patients who underwent radical gastrectomy for GC in the Gastrointestinal Surgery Department of West China Hospital, Sichuan University between January 2000 and December 2017 were reviewed. Patients who met the following criteria were included: (1) pathologically confirmed gastric adenocarcinoma; (2) underwent radical resection (R0 resection); (3) underwent TG/DG and D2/D2+ lymphadenectomy; (4) did not receive preoperative oncologic treatment; and (5) had ≥16 harvested lymph nodes. The exclusion criteria were as follows: (1) gastric remnant cancer; (2) underwent proximal gastrectomy; (3) underwent D1/D1+ lymphadenectomy; (4) received preoperative oncologic treatment; (5) number of harvested lymph nodes <16; and (6) lack of clinicopathologic data.

### 2.3. Clinicopathologic Materials

The clinicopathologic data reviewed included demographic parameters (age and sex), comorbidities, tumor location (longitudinal and cross-sectional location), macroscopic type, maximal diameter of the tumor, extent of gastrectomy, operative approach, combined organ resection, number of harvested LNs, histology type, perineural invasion, lymphovascular invasion, venous invasion, cancer nodules, pT stage, pN stage, pM stage and adjuvant chemotherapy used. Clinicopathologic features were classified according to the classification of JGCA (3rd English edition) [6]. Adenocarcinoma of the esophagogastric junction (EGJA) was defined according to the Siewert classification [18]. The TNM staging was classified according to the eighth edition of the *Cancer Staging Manual of the AJCC* [12].

### 2.4. Scope Definition of No. 12a LNs and Definition of No. 12a LND Compliance and Noncompliance

According to the classification of JGCA (3rd English edition), hepatoduodenal ligament LNs along the proper hepatic artery were defined as station 12a LNs [6]. Scope definition of No. 12a LNs in our institute were as follows: (1) upper border: the confluence of the left and right hepatic arteries; (2) lower border: the origin of the proper hepatic artery at the upper border of the pancreas; (3) lateral border: left border of the common bile duct; (4) left border: the left margin of the hepatoduodenal ligament; (5) anterior border: the anterior hepatoduodenal ligament; (6) posterior border: the anterior wall of the portal vein. Procedure of No. 12a LND was as follows. First, hepatoduodenal ligament between the lower border of liver and the duodenal bulb was fully exposed. Second, hepatoduodenal ligament between the upper border of pancreas at the origin of the proper hepatic artery and the confluence of the right and left hepatic arteries was opened at the left borderline of the common bile duct. Third, continuous perivascular sheath tissues, including all the fatty and lymphatic tissues along the proper hepatic artery and covering tissues of the anterior and medial wall of the portal vein, were en bloc dissected. A short rubber band was used to stretch the proper hepatic artery to facilitate the exposure of the portal vein. After a standard No. 12a LND procedure, the proper hepatic artery was skeletonized, and the anterior and medial wall of the portal vein was displayed. For definition of No. 12a LND compliance and noncompliance, patients were divided into the No. 12a LND compliance group or the No. 12a LND noncompliance group based on whether any LNs were harvested from station 12a. If any lymph nodes were retrieved from station 12a according to the final pathologic report, patients were assigned to the No. 12a LND compliance group; otherwise, patients were assigned to the No. 12a LND noncompliance group.

### 2.5. Follow-Up and Clinical Endpoint

All patients were followed up periodically through outpatient visits, telephone interviews, network tools and letters. The follow-up interval was every 3 to 6 months during the first 2 years postoperatively, every 6 to 12 months during the subsequent 3 years and annually thereafter. In this study, overall survival (OS) was the endpoint of interest and was calculated from the date of surgery to the date of death from any cause or the date of latest follow-up.

### 2.6. Statistical Methods

Continuous variables that fit a normal distribution were expressed as the mean ± SD and were compared using Student’s *t* test; otherwise, variables were expressed as the median [IQR] and were compared using the rank sum test. Categorical variables are expressed as numbers (%) and were compared using chi-squared tests. Kaplan-Meier curves of cumulative survival were compared using the log rank test. Logistic regression analysis was used to analyze independent predictors associated with No. 12a LN metastasis. Cox proportional hazard regression analysis was performed to identify independent predictors associated with overall survival. Those variables with a univariable *p* < 0.05 were entered into the multivariable regression model using backward stepwise variable selection. Nomograms and calibration curves of the Cox regression model and the logistic regression model were generated using the “rms” package of R version 4.1.0. The performance of the models was evaluated and validated using the concordance index (C-index) and calibrated using 1000 bootstrap samples. Calibration was evaluated using the Hosmer-Lemeshow test.

Propensity scores were calculated using a logistic regression model based on covariates, including age, sex, comorbidity, tumor location, macroscopic type, tumor size, extent of gastrectomy, operative approaches, concomitant organ resection, number of harvested LNs, histology type, perineural invasion, lymphovascular invasion, venous invasion, cancer nodules, TNM stage and adjuvant chemotherapy. A 1:1 nearest neighbor matching, without replacement, was performed with a caliper width of 5% of the standard deviation of the logit of propensity score by using the “MatchIt” package of R version 4.1.0 [19]. After PSM, the absolute standardized mean difference (SMD) was used to measure covariate balance, and an SMD threshold of 0.1 was considered substantial imbalance.

All data were analyzed using SPSS software version 24.0 (SPSS, Chicago, IL, USA) and R version 4.1.0 (http://www.r-project.org/, accessed on 28 June 2022). A two-tailed *p* < 0.05 was considered statistically significant.

## 3. Results

### 3.1. Comparison of Clinicopathologic Findings before and after PSM

As shown in Table 1, the lowest compliance rate was reported for the dissection of station 12a LNs. The No. 12a LND compliance rate of the whole study cohort was 37.1% (1035/2788). The comparison of clinicopathological parameters between the No. 12a LND compliance group and the No. 12a LND noncompliance group showed that the longitudinal location of the tumor (*p* < 0.001), distribution of macroscopic types (*p* < 0.001), tumor size (*p* < 0.001), number of harvested LNs (*p* < 0.001), perineural invasion rate (*p* < 0.001), vascular invasion rate (*p* < 0.001), venous invasion rate (*p* < 0.001), T stage distribution (*p* < 0.001), TNM stage distribution (*p* = 0.011) and proportion of patients who received adjuvant chemotherapy (*p* = 0.003) were significantly different between groups. After a 1:1 matching based on propensity scores, there were 916 patients left in each group, and the clinicopathological parameters were well balanced between the two groups (Table 2 and Figure S1).

### 3.2. Survival and Risk Factors for No. 12a LN Metastasis

**Figure 1:**
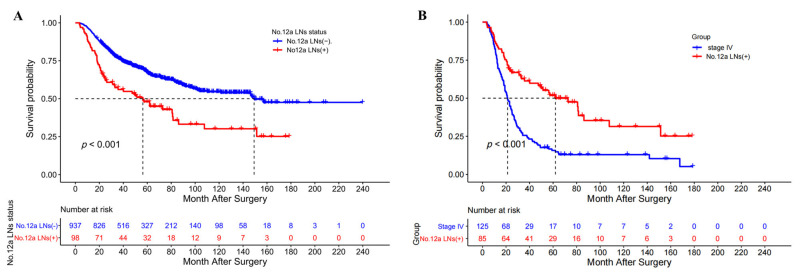
(**A**) The overall survival curves of patients with and without No. 12a LN metastasis after a D2 gastrectomy; (**B**) The overall survival curves of patients with No. 12a LN metastasis (stage IV not included) and patients with stage IV after D2 gastrectomy.

The metastasis rate of station 12a LNs in the No. 12a LND compliance group was 9.5% (98/1035). The median OS and 5-year OS rates were significantly greater in patients without No. 12a LN metastasis (149.2 vs. 56.3 months and 66.9% vs. 48%, *p* < 0.001, Figure 1A). No. 12a LN metastasis patients (stage IV not included) had a significantly better OS than stage IV patients (median OS 62.0 vs. 24.0 months, 5-year OS rate 51.8% vs. 23.0%, *p* = 0.006, Figure 1B). Logistic regression analysis revealed that age ≥ 65 years (OR = 0.461, *p* = 0.004), tumor longitudinal location (*p* = 0.017), tumor cross-sectional location (*p* = 0.048), undifferentiated type (OR 1.617, *p* = 0.045), venous invasion (OR = 2.350, *p* = 0.001), cancer nodules (OR = 2.742, *p* = 0.001), pT4 (OR = 4.056, *p* < 0.001) and distant metastasis (OR = 2.744, *p* = 0.012) were independent predictors of station 12a LN metastasis (Table 3). The nomogram of the logistic regression model for No. 12a LN metastasis is depicted in Figure S2.

### 3.3. Survival Analysis of No. 12a LND Compliance and Noncompliance

**Figure 2:**
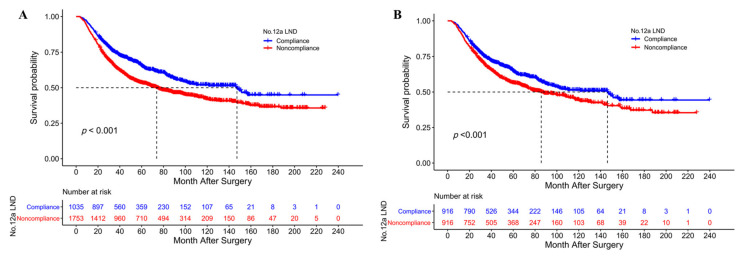
(**A**) The overall survival curves of No. 12a LND compliant and noncompliant patients after a D2 gastrectomy before PSM; (**B**) The overall survival curves of No. 12a LND compliant and noncompliant patients after a D2 gastrectomy after PSM.

Before PSM, the median OS and 5-year OS rates of patients in the No. 12a LND compliance group were significantly greater than those of patients in the No. 12a LND noncompliance group (147.4 vs. 73.8 months and 65.1% vs. 53.4%, *p* < 0.001, Figure 2A). The overall survival curves of patients with and without No. 12a LN metastasis and No. 12a LND noncompliant patients before PSM are depicted in Figure S3. After PSM, the No. 12a LND compliance group still had a better OS than the No. 12a LND noncompliance group (median OS 146.3 vs. 85.7 months, 5-year OS 64.5% vs. 56.4%, *p* < 0.001, Figure 2B). Before PSM, Cox regression analysis revealed that No. 12a LND noncompliance (HR 1.323, *p* < 0.001), age ≥ 65 years (HR 1.187, *p* = 0.003), tumor cross-sectional location (*p* = 0.029), total gastrectomy (HR 1.148, *p* = 0.032), tumor size (HR 1.028, *p* = 0.044), cancer nodules (HR 1.372, *p* < 0.001), pT stage (*p* < 0.001), pN stage (*p* < 0.001), pM stage (*p* < 0.001) and adjuvant chemotherapy (HR 0.767, *p* < 0.001) were independent prognostic factors for OS in the entire study population (Table 4). After PSM, Cox regression analysis revealed that No. 12a LND noncompliance (HR 1.353, *p* < 0.001), age ≥65 years (HR 1.177, *p* = 0.029), total gastrectomy (HR 1.225, *p* = 0.013), tumor size (HR 1.044, *p* = 0.009), cancer nodules (HR 1.441, *p* < 0.001), T stage (*p* < 0.001), N stage (*p* < 0.001), M stage (*p* = 0.010) and adjuvant chemotherapy (HR 0.807, *p* = 0.026) remained independent prognostic factors for OS in the matched cohort (Table S1).

### 3.4. Subgroup Analysis of No. 12a LND Compliance and Noncompliance

**Figure 3:**
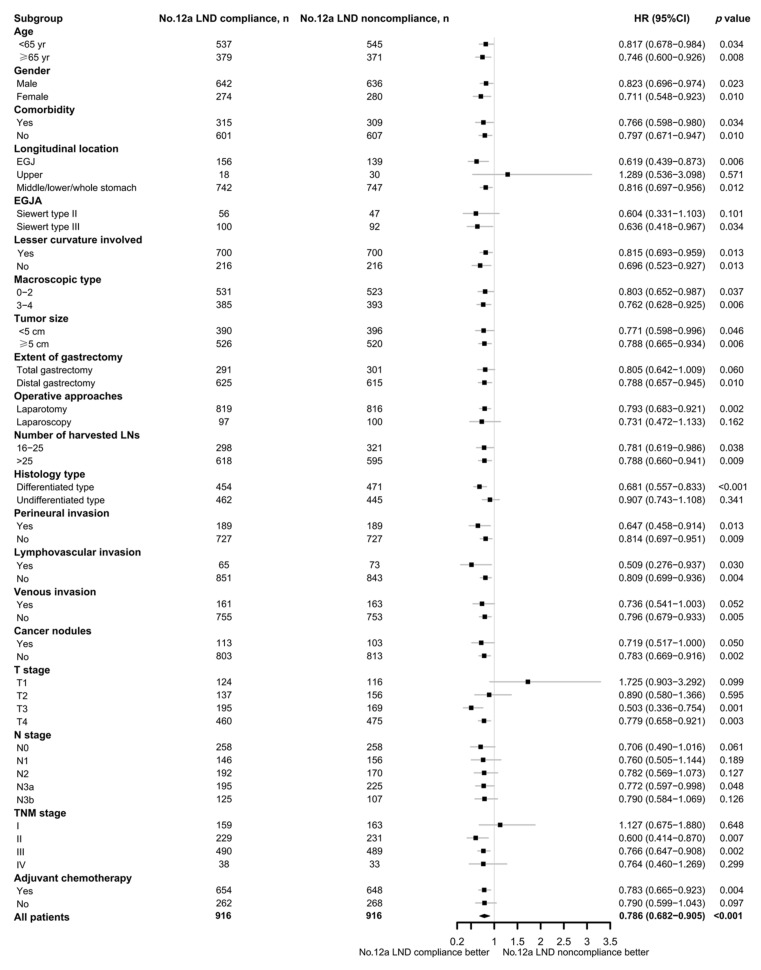
Forest plot of the subgroup analysis.

Subgroup analysis was performed in the matched cohort according to clinicopathologic parameters. It showed that compliance with No. 12a LND could bring a survival benefit for most subgroups, especially for patients with tumors involving the middle/lower third of the stomach (*p* = 0.012), with EGJA Siewert type III (*p* = 0.034), who had a tumor with or without lesser curvature involvement (both *p* < 0.05), who underwent distal gastrectomy (*p* = 0.010), who had a tumor size of <5 and ≥5 cm (both *p* < 0.05), with pT3 (*p* = 0.001) and pT4 (*p* = 0.005), with pN3a (*p* = 0.048), with stage II (*p* = 0.007) and stage III (*p* = 0.002), and who received adjuvant chemotherapy (*p* = 0.004). The detailed results of the subgroup analysis are depicted in a forest plot (Figure 3).

### 3.5. Net Survival Benefit of No. 12a LND Compliance

**Figure 4:**
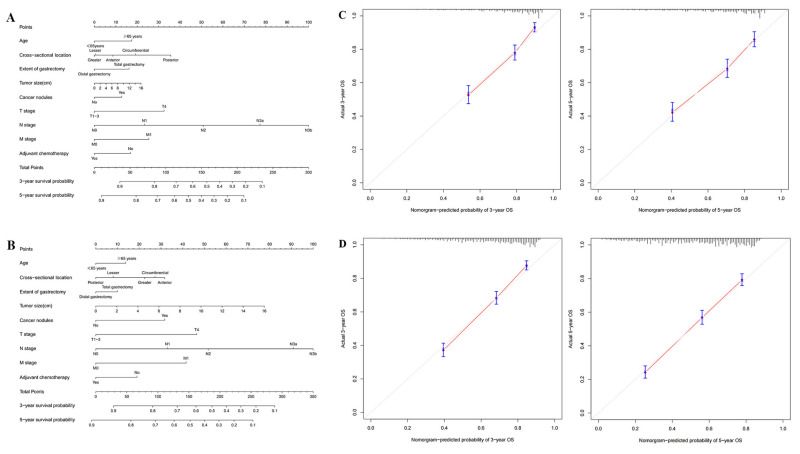
(**A**) Nomogram of No. 12a LND compliant patients; (**B**) Nomogram of No. 12a LND noncompliant patients; (**C**) Calibration curves for nomogram of No. 12a LND compliant patients; (**D**) Calibration curves for nomogram of No. 12a LND noncompliant patients.

To evaluate the net survival benefit of No. 12a LND compliance, two nomograms were established for No. 12a LND compliant patients and No. 12a LND noncompliant patients based on independent prognostic factors confirmed in the entire study population. These two nomograms were compared, and the difference between the two estimates was the expected net survival benefit from No. 12a LND compliance (Figure 4A,B). Bootstrapping with 1000 resamples demonstrated good predictive performance of the nomograms, with C-indexes of 0.732 (95% CI 0.707–0.757) for No. 12a LND compliant patients and 0.718 (95% CI 0.702–0.735) for No. 12a LND noncompliant patients. The calibration curves to predict 3- and 5-year survival probabilities among No. 12a LND compliant patients and No. 12a LND noncompliant patients also showed good consistency with the ideal predictive curves (all *p* > 0.05, Figure 4C,D).

## 4. Discussion

The role of No. 12a LN metastasis and No. 12a LND in gastric cancer is debated and is still not fully elucidated. The 5-year survival of patients with No. 12a LN metastasis has been reported to range from 5.6% to 54.4% [9,14,20,21,22]. There were inconsistencies between different guidelines in terms of whether hepatoduodenal LNs should be classified as regional LNs and be routinely removed during surgery [6,8,12,15]. The present study showed that the 5-year OS rate or No. 12a LN metastasis patients (stage IV not included) was 51.8%, which was significantly better than that of stage IV patients. Therefore, the results of this study are in accordance with the opinion that No. 12a LN metastasis should be considered regional metastasis.

In the present study, the overall No. 12a LN metastasis rate was 9.5%. The metastasis rate or No. 12a LNs varied between previous reports due to inevitable differences in clinicopathological features between studies. In fact, it has been reported that tumor location, tumor size, tumor stage, soft tissue invasion, nerve invasion, intravascular cancer emboli, macroscopic type and histological type are possible predictors of No. 12a LN metastasis [9,21,23,24]. Logistic regression in this study revealed that an older age, the tumor longitudinal location (middle or lower third involved), the tumor cross-sectional location (lesser curvature involved), the undifferentiated type, venous invasion, cancer nodules, pT4 and distant metastasis were independent predictors of station 12a LN metastasis.

The No. 12a LND compliance rate in the present study was 37.1%, which is in accordance with other studies [14,25]. This result indicated that the compliance rate or No. 12a LND is unsatisfactory in actual practice. Chen et al. [16] analyzed data from 2401 patients who underwent D2 radical gastrectomy and found that the tumor site, BMI, range of gastrectomy, previous abdominal surgery and surgery type were independent predictive factors for noncompliance with D2 lymphadenectomy. It has been reported that the endoscopic submucosal injection of carbon nanoparticle suspension or indocyanine green 1 day before surgery could improve the number of lymph node dissections performed at station 12a [26,27]. Thus, such techniques should be selectively used in patients with a high risk of No. 12a LN metastasis or a high risk of noncompliance with D2 lymphadenectomy to improve the compliance rate of station 12a LND.

In the present study, the survival analysis showed that patients with compliance with No. 12a LND achieved better OS than those without it, both before and after PSM. Further Cox regression analysis showed that noncompliance with No. 12a LND was an independent prognostic factor for OS in the entire study cohort and in the matched cohort. Based on these results, noncompliance with No. 12a LND compromised the long-term survival of patients who received TG/DG plus D2 lymphadenectomy. However, there was an apparent contradiction between the results of the present study and the results of the real-world study reported by Seo et al. [14]. A wide gap in the proportion of early-stage patients included between studies may account for this discrepancy.

To further elucidate patients who may benefit from No. 12a LND, subgroup analysis was performed in the matched cohort. It is not surprising that stage II-III patients obtained survival benefit from the compliance of No. 12a LND. Lin et al. [25] also reported that the survival of stage II and stage III LND compliant patients was significantly superior to that of LND noncompliant patients. Moreover, the compliance of No. 12a LND provided a survival benefit for patients with tumors located at the lower or middle third or the lesser curvature involved. These results matched with the reported independent predictors of station 12a LN metastasis and may be mainly due to the right gastric artery serving as a lymphatic drainage route for these tumors [9].

Another finding in the subgroup analysis was that compliance with No. 12a LND brings survival benefits to patients with tumors located at the esophagogastric junction. Patients with EGJA were further divided into Siewert type II and type III, and the results showed that patients with Siewert type III EGJA obtained survival benefits from compliance with No. 12a LND. Galizia et al. [13] analyzed data from 73 patients who underwent radical surgery for GC and reported that modified D2 (D1/D1+) lymphadenectomy conferred the same oncologic adequacy as standard D2 lymphadenectomy for tumors located in the upper or middle third of the stomach. However, the sample size in their study seems too small to draw a reliable conclusion. Yura et al. [28] surveyed 202 patients diagnosed with T2/T3 gastric cancer exclusively located in the upper third of the stomach and reported that the station 12a LN metastasis rate was 0.6% (1/162). However, another recent study from de Jongh et al. [29] reported a No. 12a LN metastasis rate of 22.7% (5/22) in patients with T3/T4 gastric cancer located in the proximal third of the stomach. In the present study, the No. 12a LN metastasis rate of patients with tumors located at the esophagogastric junction was 2.7% (5/182). A high proportion (over 40%) of pT4 tumors included in this study may explain the result that patients with Siewert type III EGJA obtained survival benefit from the compliance of No. 12a LND. Tumor size may be another risk factor for No. 12a LN metastasis in EJGA. A previous study from our institute noted that a tumor size exceeding 5.0 cm was an independent risk factor for lower perigastric lymph node metastasis [22]. Thus, further studies are warranted for the lymph node metastasis patterns in large advanced (T3/T4) gastric cancer located at the upper third of the stomach and the esophagogastric junction. According to the results of the current study, No. 12a LND should not be neglected in patients with EGJA.

There were limitations to this study. First, although PSM was used to eliminate possible confounders, selection bias, detection bias and statistical bias are unavoidable because of the inherent nature of retrospective studies. Second, the statistical results in the present study were based on data from a single center, and external validation was lacking. Third, the results of subgroup analysis may not be robust because of the small sample size of particular groups. Fourth, overall survival was the only endpoint of interest in this study due to the lack of recurrent data in the WCH-SGCPR.

In conclusion, noncompliance with station 12a LND does compromise long-term survival in patients who underwent D2 gastrectomy for gastric adenocarcinomas. The No. 12a LND procedure should be performed more carefully to improve the compliance rate.

## Figures and Tables

**Table 1:** Compliance for perigastric and extra-perigastric lymph node stations.

Lympha Node Station	Group	Number of Case, n (%)
#1	Compliance	2013 (72.2)
	Noncompliance	775 (27.8)
#2 *	Compliance	764 (83.8)
	Noncompliance	149 (16.2)
#3	Compliance	2081 (74.6)
	Noncompliance	707 (25.4)
#4sa *	Compliance	596 (65.3)
	Noncompliance	317 (34.7)
#4sb	Compliance	1736 (62.3)
	Noncompliance	1052 (37.7)
#4d	Compliance	2429 (87.1)
	Noncompliance	359 (12.9)
#5	Compliance	2154 (77.3)
	Noncompliance	634 (22.7)
#6	Compliance	2408 (83.4)
	Noncompliance	380 (13.6)
#7	Compliance	2454 (88)
	Noncompliance	334 (12)
#8a	Compliance	2168 (77.8)
	Noncompliance	620 (22.2)
#9	Compliance	2032 (72.9)
	Noncompliance	756 (27.1)
#10 *	Compliance	452 (49.5)
	Noncompliance	461 (50.5)
#11	Compliance	1542 (55.3)
	Noncompliance	1246 (44.7)
#12a	Compliance	1035 (37.1)
	Noncompliance	1753 (62.9)

* assessed in total gastrectomy cases.

**Table 2:** Details of clinicopathological features before and after 1:1 PSM.

Clinicopathological Features	Before Propensity Score Matching		*p*	After Propensity Score Matching		*p*
	No. 12a LND Compliance	No. 12a LND Noncompliance		No. 12a LND Compliance	No. 12a LND Noncompliance	
	n = 1035 (%)	n = 1753 (%)		n = 916 (%)	n = 916 (%)	
Age (years) (mean ± SD)	56.7 ± 11.3	57.4 ± 11.7	0.135	56.9 ± 11.4	56.7 ± 11.7	0.737
Gender			0.080			0.760
Male	728 (70.3)	1177 (61.8)		642 (70.1)	636 (69.4)	
Female	307 (29.7)	576 (38.2)		274 (29.9)	280 (30.6)	
Comorbidity			0.869			0.767
Yes	355 (34.3)	695 (33.9)		315 (34.4)	309 (33.7)	
No	680 (65.7)	1158 (66.1)		601 (65.6)	607 (66.3)	
Longitudinal location			<0.001			0.146
EGJ	182 (17.6)	211 (12.0)		156 (17.0)	139 (15.2)	
Upper	23 (2.2)	54 (3.1)		18 (2.0)	30 (3.3)	
Middle	105 (10.1)	236 (13.5)		94 (10.3)	117 (12.8)	
Lower	709 (68.5)	1205 (68.7)		632 (69.0)	613 (66.9)	
Whole stomach	16 (1.5)	47 (2.7)		16 (1.7)	17 (1.9)	
Cross-sectional location			0.072			0.937
Greater curvature	102 (9.9)	166 (9.5)		91 (9.9)	93 (10.2)	
Lesser curvature	673 (65.0)	1073 (61.2)		592 (64.6)	580 (63.3)	
Anterior wall	73 (7.1)	117 (6.7)		60 (6.6)	58 (6.3)	
Posterior wall	74 (7.1)	147 (8.4)		65 (7.1)	65 (7.1)	
Circumferential wall involved	113 (10.9)	250 (14.3)		108 (11.8)	120 (13.1)	
Macroscopic type			<0.001			0.705
Type 0–2	613 (59.2)	917 (52.3)		531 (58.0)	523 (57.1)	
Type 3–4	422 (40.8)	836 (47.7)		385 (42.0)	393 (42.9)	
Tumor size (cm) (median (IQR))	5 (3.4–6)	5 (4–7)	<0.001	5 (4–6)	5 (3.7–6.0)	0.775
Extent of gastrectomy			0.650			0.617
Total gastrectomy	332 (32)	581 (33.1)		291 (31.8)	301 (32.9)	
Distal gastrectomy	703 (68)	1172 (66.9)		625 (68.2)	615 (67.1)	
Operative approaches			<0.001			0.821
Laparotomy	870 (84.1%)	1613 (92%)		819 (89.4)	816 (89.1)	
Laparoscopy	165 (15.9%)	140 (8.0%)		97 (10.6)	100 (10.9)	
Combined organ resection			0.564			0.874
Yes	24 (2.2)	48 (2.5)		21 (2.3)	20 (2.2)	
No	1077 (97.8)	1062 (97.5)		915 (97.7)	907 (97.8)	
Number of harvested LNs			<0.001			0.256
16–25	298 (28.8)	936 (53.4)		298 (32.5)	321 (35.0)	
>25	737 (71.2)	817 (46.6)		618 (67.5)	695 (65.0)	
Histology type			0.554			0.427
Differentiated type	518 (50.0)	857 (48.9)		454 (49.6)	471 (51.4)	
Undifferentiated type	517 (50.0)	896 (51.1)		462 (50.4)	445 (49.1)	
Perineural invasion			<0.001			1.000
Yes	275 (26.6)	253 (14.4)		189 (20.6)	189 (20.6)	
No	760 (73.4)	1500 (85.6)		727 (79.4)	727 (79.4)	
Lymphovascular invasion			<0.001			0.479
Yes	104 (10.0)	99 (5.6)		65 (7.1)	73 (8.0)	
No	931 (90.0)	1654 (94.4)		851 (92.9)	843 (92.0)	
Venous invasion			0.002			0.903
Yes	205 (19.8)	266 (15.2)		161 (17.6)	163 (17.8)	
No	830 (80.2)	1487 (84.8)		755 (82.4)	753 (82.2)	
Cancer nodules			0.630			0.469
Yes	120 (11.6)	214 (12.2)		113 (12.3)	103 (11.2)	
No	915 (88.4)	1539 (87.8)		823 (87.7)	813 (88.8)	
T stage			<0.001			0.463
T1	153 (14.8)	206 (11.8)		124 (13.5)	116 (12.7)	
T2	173 (16.7)	258 (14.7)		137 (15.0)	156 (17.0)	
T3	229 (22.1)	275 (15.7)		199 (21.3)	169 (18.4)	
T4a	458 (44.3)	957 (54.6)		438 (49.2)	452 (49.3)	
T4b	22 (2.1)	57 (3.3)		22 (2.4)	23 (2.5)	
N stage			0.136			0.267
N0	307 (29.7)	481 (27.4)		258 (28.2)	258 (28.2)	
N1	171 (16.5)	296 (16.9)		146 (15.9)	156 (17.0)	
N2	215 (20.8)	337 (19.2)		192 (21.0)	170 (18.6)	
N3a	207 (20.0)	422 (24.1)		195 (21.3)	225 (24.6)	
N3b	135 (13.0)	217 (12.4)		125 (13.6)	107 (11.7)	
M stage			0.111			0.545
M0	997 (96.3)	1666 (95.5)		898 (95.9)	896 (95.7)	
M1	38 (3.7)	87 (4.5)		38 (4.1)	40 (4.3)	
TNM stage			0.011			0.842
IA	94 (9.1)	147 (8.4)		79 (8.6)	83 (9.1)	
IB	106 (10.2)	137 (7.8)		80 (8.7)	80 (8.7)	
IIA	118 (11.4)	148 (8.4)		96 (10.5)	89 (9.7)	
IIB	152 (14.7)	274 (15.6)		133 (14.5)	142 (15.5)	
IIIA	245 (23.7)	419 (23.9)		223 (24.3)	206 (22.5)	
IIIB	171 (16.5)	354 (20.2)		165 (18.0)	188 (20.5)	
IIIC	111 (10.7)	187 (10.7)		102 (11.1)	95 (10.4)	
IV	38 (3.7)	87 (5.0)		38 (4.1)	33 (3.6)	
Adjuvant chemotherapy			0.003			0.757
Yes	740 (71.5)	1157 (66.0)		654 (71.4)	648 (70.7)	
No	295 (28.5)	596 (34.0)		262 (28.6)	268 (29.3)	

**Table 3:** Logistic regression analysis of independent risk factors for No. 12a LN metastasis.

Factors	No. 12a LN Metastasis Case, n (%)	Univariate Analysis		Multivariate Analysis	
		OR (95% CI)	*p*	OR (95% CI)	*p*
Age			0.018		0.004
<65 years	71 (11.5)	Reference		Reference	
≥65 years	27 (6.4)	0.502 (0.284–0.887)	0.018	0.461 (0.274–0.777)	0.004
Gender			0.829		
Female	30 (9.8)	Reference			
Male	68 (9.3)	0.951 (0.605–1.495)	0.829		
Longitudinal location			0.024		0.017
EGJ	5 (2.7)	Reference		Reference	
Upper	2 (8.7)	3.371 (0.615–18.475)	0.161	2.443 (0.412–14.482)	0.325
Middle	10 (9.5)	3.726 (1.238–11.218)	0.019	3.998 (1.238–12.914)	0.021
Lower	78 (11.0)	4.376 (1.745–10.974)	0.002	5.061 (1.932–12.261)	0.001
Whole stomach	3 (18.8)	8.169 (1.755–38.037)	0.007	2.450 (0.485–12.370)	0.278
Cross-sectional location			0.003		0.048
Greater curvature	4 (3.9)	Reference		Reference	
Lesser curvature	65 (9.7)	2.619 (0.933–7.351)	0.067	3.598 (1.202–10.772)	0.022
Anterior wall	5 (6.8)	1.801 (0.467–6.954)	0.393	2.392 (0.567–10.097)	0.235
Posterior wall	3 (4.1)	1.035 (0.225–4.770)	0.965	1.298 (0.264–6.379)	0.748
Circumferential involvement	21 (18.6)	5.592 (1.850–16.909)	0.002	4.622 (1.413–15.121)	0.011
Macroscopic types			<0.001		0.140
Type 0–2	40 (6.5)	Reference		Reference	
Type 3–4	58 (13.7)	2.283 (1.494–3.487)	<0.001	1.444 (0.886–2.355)	0.140
Tumor size			0.001		0.443
<5 cm	30 (6.3)	Reference		Reference	
≥5 cm	68 (12.2)	2.068 (1.321–3.237)	0.001	1.233 (0.723–2.103)	0.443
Histological type			0.002		0.045
Differentiated type	34 (6.6)	Reference		Reference	
Undifferentiated type	64 (12.3)	2.011 (1.301–3.108)	0.002	1.617 (1.012–2.583)	0.045
Perineural invasion			0.637		
No	70 (9.2)	Reference			
Yes	28 (10.2)	1.117 (0.704–1.773)	0.637		
Lymphovascular invasion			0.957		
No	88 (9.5)	Reference			
Yes	10 (9.6)	1.019 (0.512–2.028)	0.957		
Venous invasion			<0.001		0.001
No	64 (7.7)	Reference		Reference	
Yes	34 (16.6)	2.380 (1.521–3.724)	<0.001	2.350 (1.422–3.884)	0.001
Cancer nodules			<0.001		0.001
No	72 (7.9)	Reference		Reference	
Yes	26 (21.7)	3.238 (1.971–5.321)	<0.001	2.742 (1.539–4.885)	0.001
Depth of tumor			<0.001		<0.001
T1–3	21 (3.8)	Reference		Reference	
T4	77 (16.0)	4.859 (2.948–8.007)	<0.001	4.056 (2.376–6.926)	<0.001
M stage			<0.001		0.012
M0	85 (8.8)	Reference		Reference	
M1	13 (34.2)	5.579 (2.754–11.304)	<0.001	2.744 (1.250–6.027)	0.012

**Table 4:** Univariate and multivariate survival analysis in this study by Cox proportion hazard model before PSM.

Features	Univariate Analysis			Multivariate Analysis		
	HR	95% CI	*p*	HR	95% CI	*p*
No. 12a LND, noncompliance	1.422	1.260–1.605	<0.001	1.323	1.171–1.496	<0.001
Age, ≥65 years	1.177	1.054–1.316	0.004	1.187	1.060–1.330	0.003
Gender, male	1.001	0.889–1.127	0.985			
Comorbidity, yes	0.911	0.811–1.023	0.116			
Longitudinal location			<0.001			0.580
EGJ	Reference			Reference		
Upper	1.286	0.914–1.810	0.149	0.870	0.609–1.242	0.240
Middle	0.902	0.729–1.115	0.166	1.070	0.858–1.334	0.669
Lower	0.813	0.813–0.953	0.011	1.064	0.844–1.340	0.893
Whole stomach	2.279	1.650–3.148	<0.001	1.235	0.874–1.745	0.223
Cross-sectional location			<0.001			0.029
Greater curvature	Reference			Reference		
Lesser curvature	0.846	0.702–1.020	0.080	0.906	0.751–1.039	0.304
Anterior wall	0.920	0.700–1.210	0.553	1.134	0.859–1.497	0.375
Posterior wall	0.844	0.648–1.100	0.211	0.969	0.742–1.265	0.817
Circumferential involvement	1.585	1.278–1.956	<0.001	1.141	0.916–1.420	0.239
Extent of gastrectomy, total gastrectomy	1.632	1.458–1.827	<0.001	1.148	1.012–1.302	0.032
Combined resection, yes	1.583	1.151–2.178	0.005	1.021	0.738–1.413	0.886
Number of harvested LNs, 16–25	1.039	0.930–1.161	0.498			
Macroscopic types, type 3–4	2.085	1.865–2.331	<0.001	1.130	0.987–1.292	0.076
Tumor size	1.173	1.151–1.195	<0.001	1.028	1.001–1.055	0.044
Histological finding, undifferentiated type	1.045	0.936–1.166	0.434			
Perineural invasion, yes	1.085	0.925–1.272	0.318			
Lymphovascular invasion, yes	1.186	0.943–1.490	0.145			
Venous invasion, yes	1.531	1.329–1.764	<0.001	1.102	0.954–1.274	0.200
Cancer nodules, yes	2.239	1.937–2.588	<0.001	1.372	1.180–1.595	<0.001
T stage			<0.001			<0.001
T1	Reference			Reference		
T2	1.790	1.326–2.418	<0.001	1.428	1.053–1.936	0.022
T3	1.972	1.466–2.652	<0.001	1.321	0.969–1.802	0.078
T4	4.746	3.684–6.114	<0.001	2.312	1.750–3.055	<0.001
N stage			<0.001			<0.001
N0	Reference			Reference		
N1	1.499	1.217–1.846	<0.001	1.404	1.131–1.741	<0.001
N2	2.259	1.874–2.722	<0.001	1.870	1.530–2.285	<0.001
N3a	3.904	3.283–4.641	<0.001	2.906	2.389–3.535	<0.001
N3b	5.847	4.853–7.045	<0.001	3.446	2.771–4.285	<0.001
M stage, M1	3.476	2.856–4.231	<0.001	1.504	1.204–1.879	<0.001
Adjuvant chemotherapy, yes	1.292	1.145–1.457	<0.001	0.767	0.666–0.882	<0.001

## Data Availability

The raw data and the original procedure video from this study are available from the corresponding author upon request.

## Supplementary Materials

The following supporting information can be downloaded at: https://www.mdpi.com/article/10.3390/cancers15030749/s1, Figure S1: Standardized mean differences of clinicopathological parameters before and after PSM; Figure S2: Nomogram of the logistic regression model for No. 12a LN metastasis; Figure S3: The overall survival curves of patients with and without No. 12a LN metastasis and No. 12a LND noncompliant patients before PSM; Table S1: Univariate and multivariate survival analysis in this study by Cox proportion hazard model after PSM.
