# Supplementary material for: Is No. 12a Lymph Node Dissection Compliance Necessary in Patients Who Undergo D2 Gastrectomy for Gastric Adenocarcinomas? A Population-Based Retrospective Propensity Score Matching Study
Source: Cancers (Basel). 2023 Jan 25;15(3):749. doi: 10.3390/cancers15030749 (PMC9913786; doi:10.3390/cancers15030749)
Supplement: Supplementary file 1 [file cancers-15-00749-s001.zip › Table S1.pdf]

Table S1. Univariate and multivariate survival analysis in this study by Cox proportion hazard model after PSM

Features	Univariate analysis			Multivariate analysis		
	HR	95%CI	*p* value	HR	95%CI	*p* value
No.12a LND, noncompliance	1.272	1.105-1.465	**0.001**	1.353	1.173-1.560	**<0.001**
Age, ≥65 years	1.186	1.029-1.367	**0.018**	1.177	1.017-1.362	**0.029**
Gender, male	1.010	0.866-1.177	0.904			
Comorbidity, yes	0.867	0.746-1.007	0.061			
Longitudinal location			**<0.001**			0.111
EGJ	Reference			Reference		
Upper	1.134	0.722-1.780	0.586	0.772	0.484-1.231	0.082
Middle	0.912	0.700-1.188	0.495	1.283	0.974-1.398	0.276
Lower	0.777	0.642-0.941	0.010	1.321	0.990-1.763	0.181
Whole stomach	2.149	1.380-3.346	0.001	1.154	0.717-1.857	0.768
Cross-sectional location			**<0.001**			0.057
Greater curvature	Reference			Reference		
Lesser curvature	0.757	0.602-0.951	0.017	0.822	0.653-1.035	0.095
Anterior wall	0.754	0.529-1.075	0.119	0.984	0.685-1.413	0.931
Posterior wall	0.820	0.587-1.147	0.247	0.990	0.706-1.387	0.951
Circumferential wall involved	1.397	1.070-1.825	0.014	1.070	0.815-1.405	0.625
Gastrectomy, total gastrectomy	1.667	1.443-1.925	**<0.001**	1.225	1.044-1.436	**0.013**
Combined resection, yes	1.523	0.987-2.351	0.057			
Number of harvested LNs, 16-25	0.999	0.863-1.156	0.987			
Macroscopic types, type 3-4	1.997	1.734-2.299	**<0.001**	1.109	0.937-1.312	0.179
Tumor size (cm)	1.176	1.147-1.206	**<0.001**	1.044	1.011-1.079	**0.009**
Histological finding, undifferentiated type	1.033	0.898-1.188	0.652			
Perineural invasion, yes	1.290	1.067-1.558	**0.008**	1.032	0.842-1.265	0.667

Lymphovascular invasion, yes	1.116	0.832-1.496	0.464			
Venous invasion, yes	1.717	1.444-2.041	**<0.001**	1.132	0.946-1.353	0.199
Cancer nodules, yes	2.415	2.011-2.900	**<0.001**	1.441	1.186-1.752	**<0.001**
T stage			**<0.001**			**<0.001**
T1	Reference			Reference		
T2	2.007	1.376-2.926	<0.001	1.518	1.034-2.227	0.033
T3	2.251	1.554-3.261	<0.001	1.454	0.984-2.149	0.060
T4	4.862	3.513-6.729	<0.001	2.335	1.634-3.337	<0.001
N stage			**<0.001**			**<0.001**
N0	Reference			Reference		
N1	1.442	1.104-1.883	0.007	1.282	0.974-1.688	0.077
N2	2.166	1.707-2.749	<0.001	1.758	1.361-2.269	<0.001
N3a	3.621	2.907-4.509	<0.001	2.600	2.031-3.328	<0.001
N3b	5.996	4.738-7.588	<0.001	3.558	2.691-4.704	<0.001
M stage, M1	3.493	2.681-4.551	**<0.001**	1.490	1.099-2.021	**0.010**
Adjuvant chemotherapy, yes	1.435	1.222-1.685	**<0.001**	0.807	0.669-0.974	**0.026**
