# Supplementary figures and images for: Is No. 12a Lymph Node Dissection Compliance Necessary in Patients Who Undergo D2 Gastrectomy for Gastric Adenocarcinomas? A Population-Based Retrospective Propensity Score Matching Study
Source: Cancers (Basel). 2023 Jan 25;15(3):749. doi: 10.3390/cancers15030749 (PMC9913786; doi:10.3390/cancers15030749)

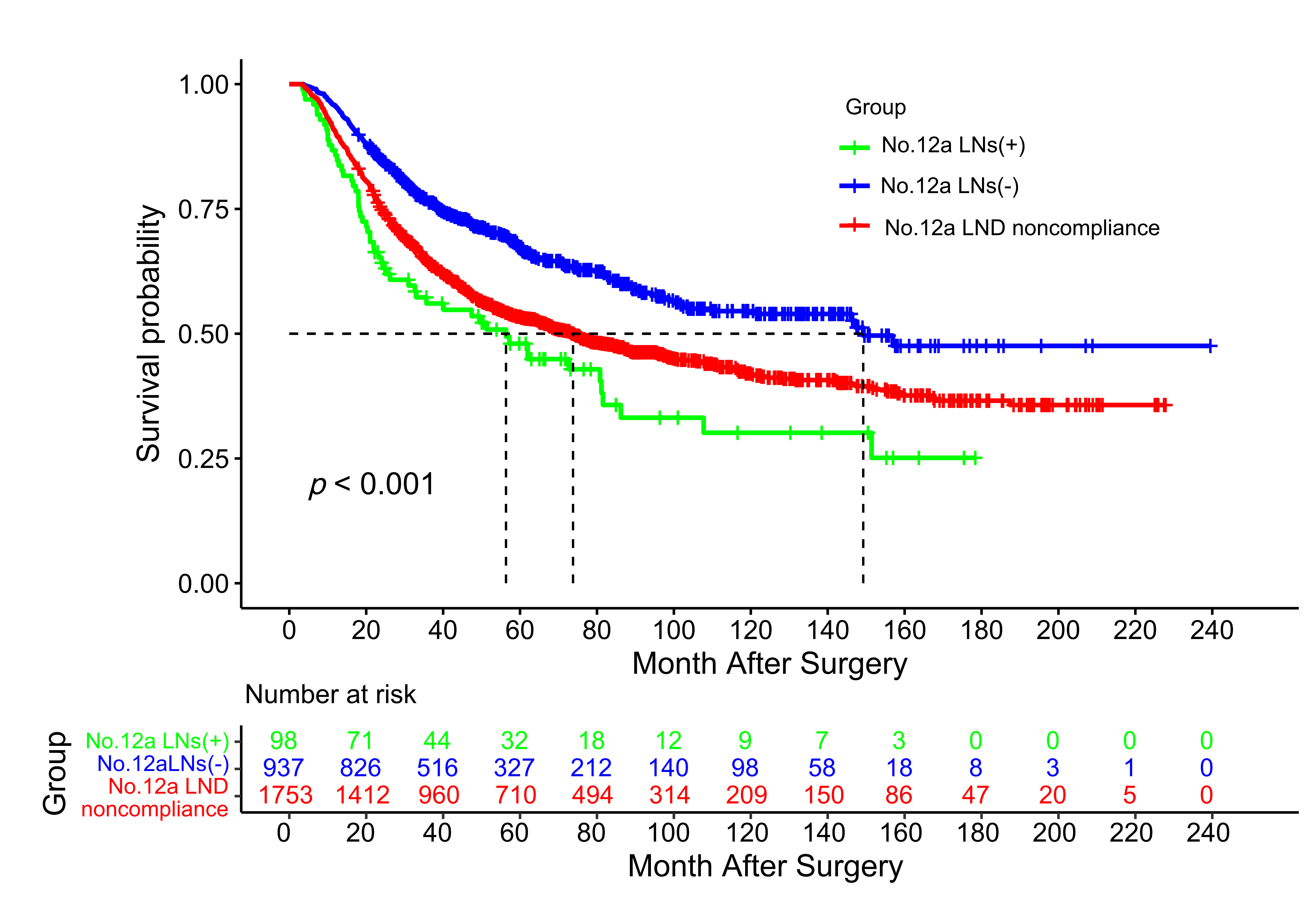

Supplement: Supplementary file 1 [file cancers-15-00749-s001.zip › Figure S3.tif]
